# Impact of the blooming artefact on dental implant dimensions in 13 cone-beam computed tomography devices

**DOI:** 10.1186/s40729-021-00347-6

**Published:** 2021-07-14

**Authors:** Victor Aquino Wanderley, Karla de Faria Vasconcelos, Andre Ferreira Leite, Ruben Pauwels, Sohaib Shujaat, Reinhilde Jacobs, Matheus L. Oliveira

**Affiliations:** 1 OMFS-IMPATH Research Group, Department of Imaging and Pathology, Faculty of Medicine, KU Leuven and Oral and Maxillofacial Surgery, University Hospitals Leuven, Leuven, Belgium; 2 Division of Oral Radiology, Department of Oral Diagnosis, Piracicaba Dental School, University of Campinas, Piracicaba, SP Brazil; 3 Department of Dentistry, Faculty of Health Sciences University of Brasília, Brasília, Brazil; 4 Aarhus Institute of Advanced Studies, Aarhus University, Aarhus, Denmark; 5 Department of Mechanical Engineering, Catholic University of Leuven, Leuven, Belgium; 6 Department of Dental Medicine, Karolinska Institutet, Stockholm, Sweden

**Keywords:** Artefacts, Cone-beam computed tomography, Dental implants

## Abstract

**Background:**

The purpose of this study was to objectively assess dimensional alteration (blooming artefact) on dental implant using 13 cone-beam computed tomography (CBCT) devices adjusted to device-specific scanning protocols and to assess whether subjective adjustment of brightness and contrast (B&C) could alter its visualization.

**Methods:**

An anthropomorphic phantom containing a dental implant was scanned in 13 CBCT devices adjusted to three scanning protocols: medium-FOV standard resolution, small-FOV standard resolution, and small-FOV high resolution. The diameter of the implant was measured at five levels, averaged, and compared with those from a reference standard industrial CT image. B&C adjustments were performed and measurements were repeated. The intraclass correlation coefficient assessed the reliability of the measurements and general linear mixed models were applied for multiples comparisons at a 95% confidence interval.

**Results:**

Implant diameter obtained from small-FOV high-resolution protocols in most CBCT devices was not significantly different when compared to that from the reference (*p* > 0.05). For standard protocols, significant dimensional alteration of the implant ranging from 23 to 34% (0.67 to 1.02 mm) was observed in 9 CBCT devices for small-FOV scanning (*p* < 0.05), and in 8 CBCT devices for medium-FOV scanning, implant dimensional alteration ranged significantly from 21 to 35% (0.62 to 1.04 mm). After B&C adjustments, dimensional alteration was reduced for several of the CBCT devices tested (*p* < 0.05).

**Conclusions:**

The visualization of the implant dimensional alteration differed between CBCT devices and scanning protocols with an increase in diameter ranging from 0.27 to 1.04 mm. For most CBCT devices, B&C adjustments allowed to reduce visualization of implant blooming.

## Introduction

Despite scientific evidence regarding the wide applicability of cone-beam computed tomography (CBCT) for diagnosis and treatment planning [1], the presence of artefacts generated by high-density materials may seriously jeopardize image quality [2]. Considering the frequent use of CBCT in implant dentistry, the presence of dental implants within the scanned volume may generate artefacts compromising the final image [3]. One of these artefacts may compromise the dimensional accuracy of high-density materials, such as titanium and zirconium dental implants, in the reconstructed CBCT image [4–6] and was initially referred to as “blooming” to describe overestimated calcified atheromatous plaques in CT images [7].

Nowadays, there is a large number of CBCT devices in the market offering several protocols with varying exposure and/or reconstruction parameters [8]. Previous studies have indicated that artefacts, including the blooming artefact type, can be partially affected by different scanning protocols [2, 5, 9]. A study on implant segmentation showed that, for most CBCT devices, blooming extends approximately one voxel in each direction, but for some devices or scanning protocols the distortion is more severe [10].

Because the delineation between a dental implant and the surrounding bone may be faded, previous studies have demonstrated the positive influence of image brightness and contrast (B&C) adjustments on the dimensional representation of different high-density materials [11, 12]; the visualization of such adjustments may vary amongst CBCT devices, depending on the severity of the artefact and the effective contrast resolution.

A gap of knowledge still exists regarding blooming artefact assessment and visualization on the wide variety of CBCT devices. Such artefact significantly affects the evaluation of the surrounding structures of an implant and might impair the analysis of peri-implant bone, osseointegration, buccal bone, bone grafting, and bone crater. Therefore, the aims of the present study were (a) to objectively assess blooming artefact (i.e., dimensional alteration) around a dental implant using 13 different CBCT devices with device-specific scanning protocols and (b) to assess whether manual adjustment of B&C could alter blooming artefact visualization.

## Materials and method

### Imaging phantom

**Fig. 1:**
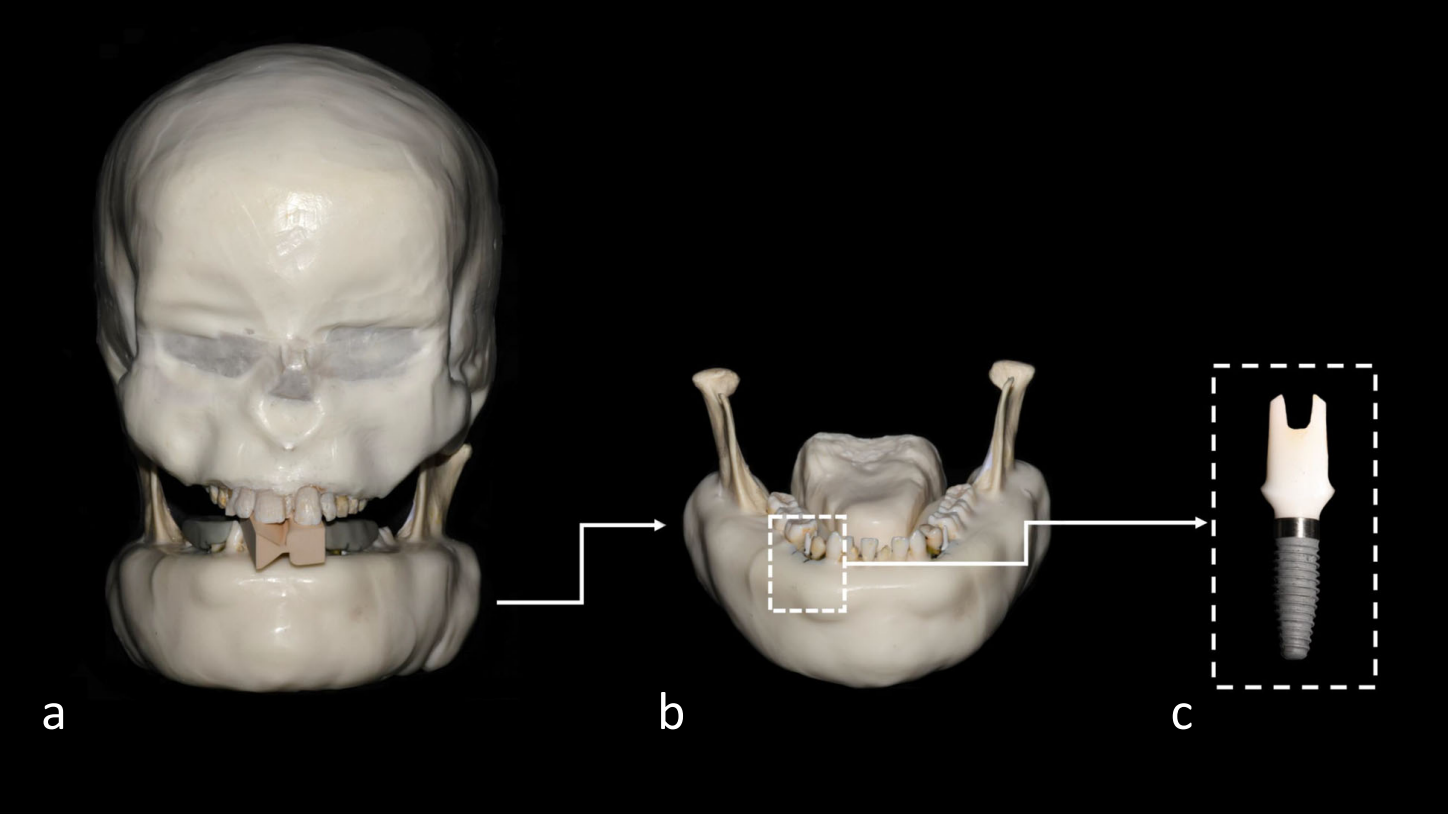
Photographs of the imaging phantom. **a** Frontal view. **b** Frontal view of the mandible showing the tongue and implant of interest in place. **c** Magnified view of the studied implant

This experimental study was designed and approved according to the regulations of the Belgian National Council for Bioethics Research Committee (protocol number NH019 2019-09-03) and the Helsinki Declaration [13]. An anthropomorphic phantom composed of a dentate dry human skull and mandible covered with Mix-D, a material to simulate soft tissue attenuation [14, 15], was used (Fig. 1). A tongue model was also created from Mix-D and fixed in the lingual region of the mandible. Then, the mandibular second premolars from both sides were carefully extracted and replaced with a titanium implant (10 mm in length and a platform with a 3.5-mm diameter) with internal tri-channel connection, model tapered, partial machined collar (PMC), and narrow platform (NP) (Nobel Biocare, Zurich, Switzerland).

### CBCT scanning

The imaging phantom was scanned using 13 CBCT devices. Three scanning protocols of different field of view (FOV) sizes and spatial resolutions were established for each CBCT device to assess blooming artefacts, as follows: medium FOV standard resolution, small FOV standard resolution, and small FOV high-resolution. For each device, clinically applicable settings were selected. Whereas the protocols were matched between CBCT devices, especially considering the FOV size, the tube voltage (kV), tube current (mA), and exposure time (s) are specific for each device. The small-FOV standard and small-FOV high-resolution protocols were not available, respectively, in two and one CBCT devices.

The studied CBCT devices were as follows: 3D Accuitomo 170 (J. Morita, Kyoto, Japan), Veraview X800 (J. Morita, Kyoto, Japan), Vera view epocs 3D R100 (J. Morita, Kyoto, Japan), X1 (3Shape, Copenhagen, Denmark), New Tom VGi evo (Cefla Dental Group, Imola, Italy), OP 3D Pro (Instrumentarium, Tuusula, Finland), OP 3D (Instrumentarium, Tuusula, Finland), CS 9300 (Carestream, Rochester, NY, USA), ProMax 3D Max (Planmeca, Helsinki, Finland), Orthophos SL 3D (Dentsply Sirona, Charlotte, NC, USA), Spectral Dental CBCT (UEG Medical, Shanghai, China), KaVo 3D eXam+ (Kavo Kerr, Charlotte, NC, USA), and PaX-i3D Green (Vatech, Seoul, Republic of Korea). Table 1 shows the scanning parameters for all CBCT devices and their corresponding codes.

**Table 1:** Studied CBCT devices with corresponding codes and scanning parameters

CBCT devices	Code	Medium FOV standard						Small FOV standard						Small FOV high-resolution					
		FOV (cm)	kV	mA	Voxel size (mm)	Exposure time (s)	DAP (mGy cm^**2**^)	FOV (cm)	kV	mA	Voxel size (mm)	Exposure time (s)	DAP (mGy cm^**2**^)	FOV (cm)	kV	mA	Voxel size (mm)	Exposure time (s)	DAP (mGy cm^**2**^)
3D Accuitomo 170	A	8 × 8	90	5	0.125	30.8	2310	4 × 4	90	5	0.125	17.5	402	4 × 4	90	5	0.08	30.8	706
Veraview X800	B	8 × 8	100	8	0.125	17.9	2112	4 × 4	100	8	0.125	17.9	596	4 × 4	100	8	0.08	17.9	589
X1	C	8 × 8	90	12	0.15	8.73	773	4 × 4	90	12	0.15	8.73	231	4 × 4	90	12	0.1	11.7	309
NewTom VGi evo	D	8 × 8	110	3	0.125	4.32	329	5 × 5	110	3	0.125	4.32	148	5 × 5	110	3	0.1	4.32	148
OP 3D Pro	E	8 × 8	90	7	0.2	6	899	5 × 5	90	7	0.125	6	452	5 × 5	90	9	0.08	8.7	775
OP 3D	F	6 × 9	95	3	0.2	20	940	5 × 5	95	4	0.125	10	582	5 × 5	95	4	0.08	20	1165
CS 9300	G	8 × 8	90	4	0.18	8	455	5 × 5	90	4	0.2	20	572	5 × 5	90	4	0.09	20	572
ProMax 3D Max	H	10 × 10	96	7	0.15	15	668	6 × 6	96	7	0.15	15	657	6 × 6	96	12	0.07	15	1155
Orthophos SL 3D	I	8 × 8	85	6	0.16	14	657	5 × 5	85	10	0.16	4.3	214	5 × 5	85	6	0.08	14	421
Spectral Dental CBCT	J	9 × 9	110	8	0.2	17	462	6 × 6	110	8	0.2	17	200	6 × 6	110	8	0.07	27	527
Veraviewepocs 3D R100	K	8 × 8	90	8	0.125	9.4	1780	4 × 4	90	8	0.125	9.4	525	*	*	*	*	*	*
KaVo 3D eXam+	L	8 × 8	120	5	0.125	7.4	500	*	*	*	*	*	*	*	*	*	*	*	*
Pax-i3D Green	M	8 × 8	104	5	0.120	11	409	*	*	*	*	*	*	*	*	*	*	*	*

*FOV* Field of view, *kV* kilovoltage, *mA* milliamperage, *DAP* Dose-area product indicated by the manufacturer

*Protocol not available

In order to acquire a reference image, the same phantom was scanned using a Nikon XT H 225 industrial computed tomography (CT) scanner (Nikon Metrology Inc., Brighton, MI, USA) adjusted to the following parameters: 200 kV, 350 μA, 2.5 mm Cu filtration, 8 × 8 cm FOV, and 40 μm voxel size. This imaging modality was used as reference for delivering better image quality due to its relative high voltage and small focal spot, which enables a more adequate assessment of the volume, diameter, screw thread morphology, and implant design.

### Image registration

To assess blooming artefact on the same implant level amongst the different CBCT devices, a voxel-based registration using the maximization of mutual information metric was applied, having the industrial CT image as a fixed reference to align each CBCT image. The mutual information method measures the statistical dependence or information redundancy between image intensities of corresponding voxels in both images, which is assumed to be maximal if images are geometrically aligned [16]. The registration procedure was performed using Amira 2019 (Thermo Fisher Scientific, Waltham, USA/Zuse Institute Berlin, Germany). After image registration, the accuracy of superimposition was visually confirmed by comparing fine anatomical structures in the trabecular and cortical bone, as well as in the adjacent teeth.

### Image analysis

**Fig. 2:**
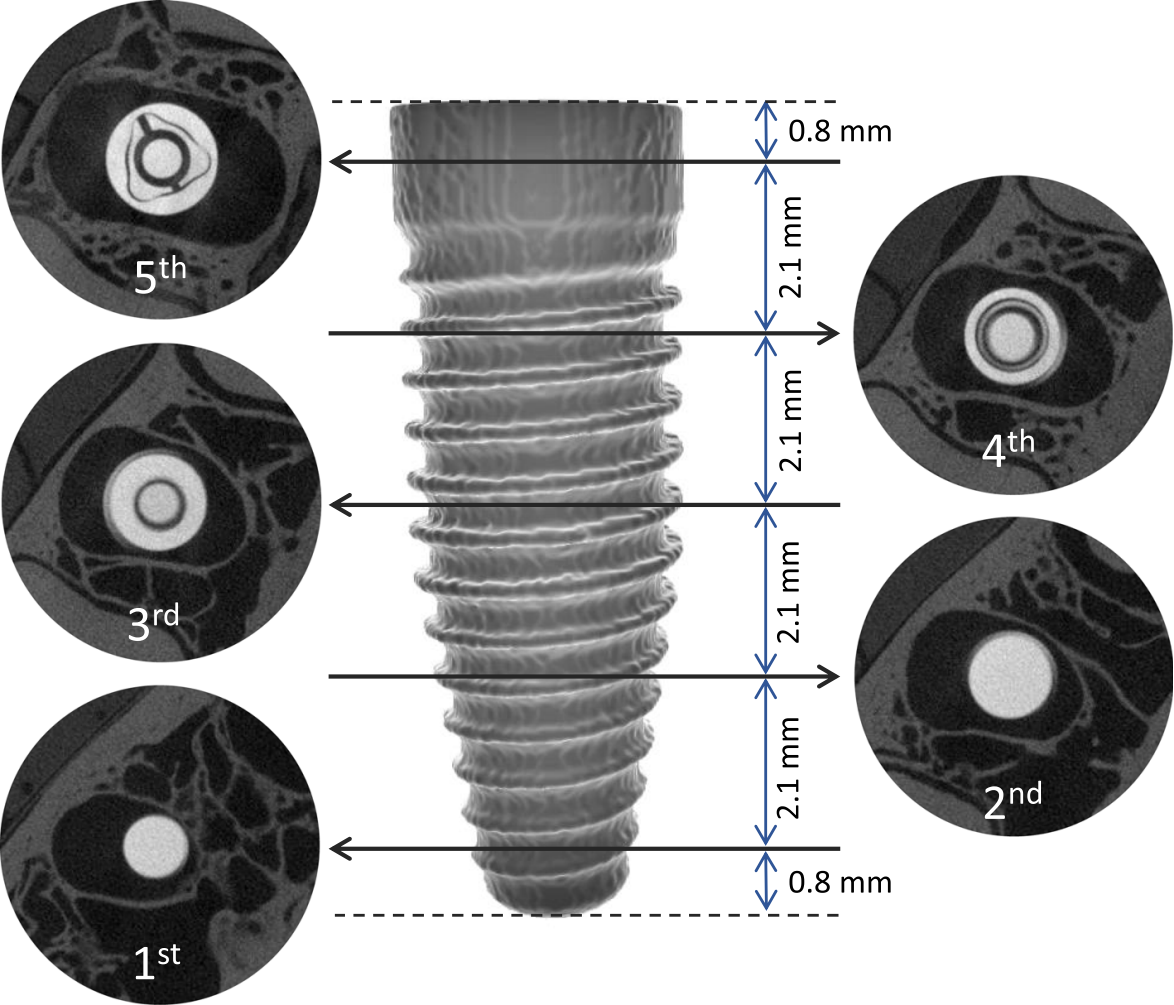
Illustration of the five implant levels and the corresponding axial slices from which the diameter was measured and averaged. The illustrated images were acquired in the industrial CT scanner (reference image)

To assess blooming artefacts on different CBCT images and scanning protocols, the diameter of the implant was measured from left to right at five axial levels including the screw thread, averaged, and compared with the reference image. Image registration assured that the measurements were performed on corresponding slices among all CBCT volumes. As illustrated in Fig. 2, the first and last levels were located 0.8 mm from the upper and lower limits of the implant and the central levels were 2.1 mm from each other. Two oral radiologists with more than 5 years of clinical experience with CBCT performed all measurements using ImageJ software version 1.50b (National Institutes of Health, Bethesda, MD, USA) in a low-light environment with a 24.1-in. flat screen monitor with a 1920 × 1080-pixel resolution (MDRC-2124, Barco N.V., Kortrijk, Belgium). The B&C level for each CBCT image was determined by pressing the “auto” button in the ImageJ software only once.

Subsequently, subjective B&C adjustments were performed in all CBCT images to visualize the abutment screw inside the implant as sharply as possible. Then, the diameter of the implant was measured again following the aforementioned method for further comparison with the original images.

After 30 days, all implant diameter measurements from the original images and B&C-adjusted images were repeated to assess reproducibility.

### Statistical analysis

The intraclass correlation coefficient (ICC) was performed to test the intra- and inter-rater reproducibility of the implant diameter measurements.

Statistical modelling and analysis were performed in S-Plus for Linux 8.0 (Tibco Software, Palo Alto, CA, USA). General linear mixed models (GLM) were applied for multiple comparisons of the diameter of the implant obtained from the reference image with those obtained from each CBCT device, scanning protocol, and implant level at a 95% confidence interval.

The same GLM was used to compare the diameter of the implant obtained from the original CBCT images with those from the CBCT images subjected to B&C adjustments.

For a better interpretation of the outcomes, absolute (in millimetres) and relative (in percentage) discrepancy values between implant diameter from the reference image and the CBCT image from all CBCT devices and scanning protocols were obtained.

## Results

The implant diameter measurements showed an excellent intra-rater (ICC = 0.85 to 0.97) and inter-rater (ICC = 0.87) reproducibility for all CBCT devices and scanning protocols.

**Fig. 3:**
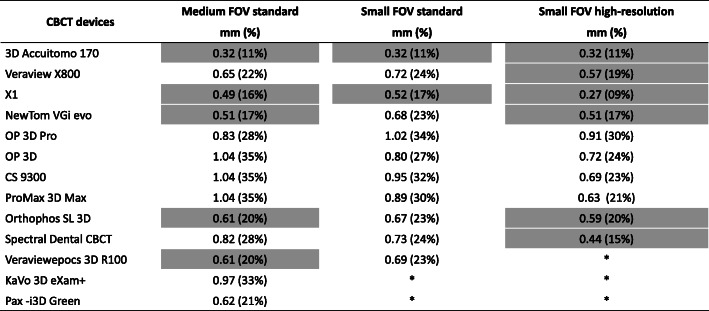
Blooming artefact in millimetres and percentage increase of implant diameters compared with reference image for all studied CBCT devices and scanning protocols. The cells highlighted with light grey indicate that the implant measurements did not differ significantly from those of the reference image. Asterisks indicate the unavailable protocols

**Fig. 4:**
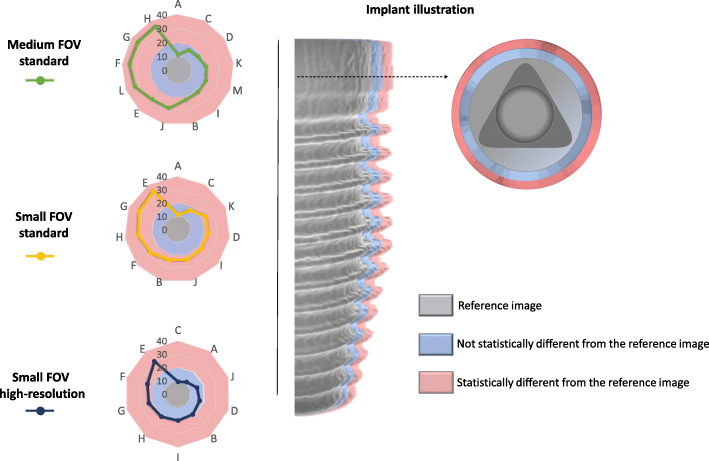
Left: Radar charts of the discrepancy (in percentage) of the diameter between the reference image and each CBCT device for the three scanning protocols. Right: An illustration of an implant (grey) with non-significant (blue) and significant (pink) expressions of the blooming artefact

Most of the small FOV high-resolution protocols did not show a significant increase (*p* > 0.05) in implant diameter compared with the reference industrial CT image, ranging from 0.27 mm (9%) to 0.59 mm (20%) (Fig. 3). For the small FOV standard protocol, only two CBCT devices (A and C) did not differ significantly from the reference image, ranging from 0.32 mm (11%) to 0.52 mm (17%). Regarding the medium FOV standard protocol, devices A, C, D, I, and K (as coded in Table 1) were not statistically different from the reference image and ranged from 0.32 mm (11%) to 0.61 mm (17%). In devices A and C, the blooming artefact did not significantly affect the implant diameter from the reference image for all studied scanning protocols. Devices E, F, G, H, L, and M differed significantly (*p* < 0.05) from the reference image, irrespective of the scanning protocol, ranging from 0.62 mm (21%) to 1.04 mm (35%). Fig. 4 is a schematic representation of the expression of the blooming artefact in implant diameter for all CBCT devices and scanning protocols.

**Fig. 5:**
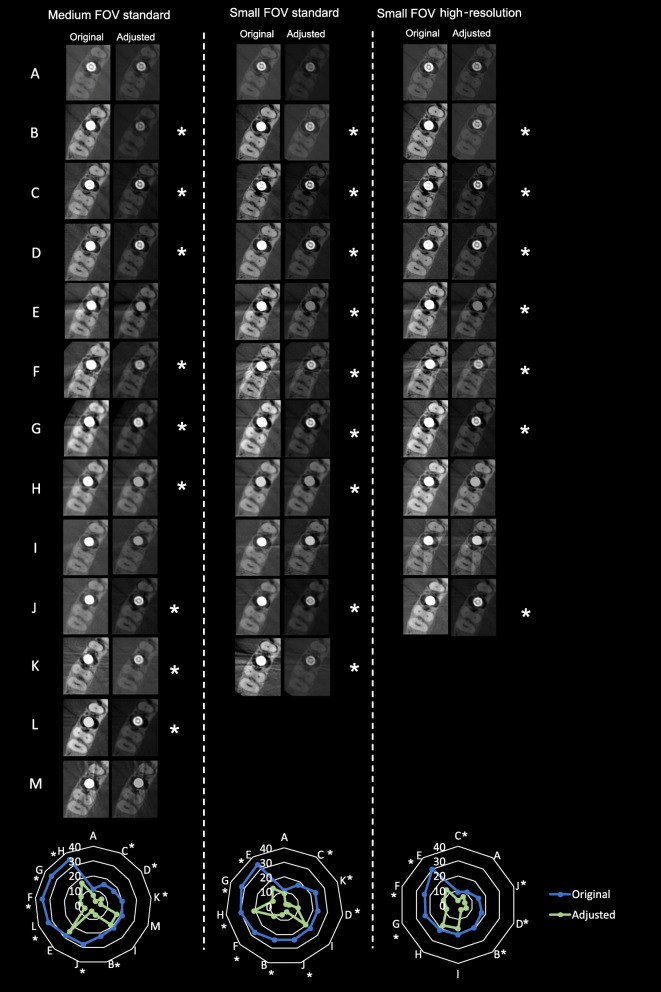
Representative cropped axial slices of the original and B&C-adjusted images of all CBCT devices and scanning protocols. Asterisks indicate significant reduction of the negative impact of the blooming artefact after B&C adjustments. At the bottom of the figure, radar charts show the discrepancy (in percentage) of the implant diameter between the original and adjusted images of the CBCT devices for each device and scanning protocol

After B&C adjustments, a statistically significant reduction in overestimated implant diameter was found in most CBCT devices (*p* < 0.05): 9 out of 13 devices for the medium FOV standard protocol, 9 out of 11 devices for the small FOV standard protocol, and 7 out of 10 devices for the small FOV high-resolution protocol. For devices A and I, the differences in implant diameter were not statistically significant between the original and B&C-adjusted images irrespective of the scanning protocol (*p* > 0.05) (Fig. 5). This difference was not statistically significant (*p* > 0.05) either for devices E and M in the medium FOV standard protocol and for device H in the small FOV high-resolution protocol. No statistically significant difference (*p* > 0.05) was observed when comparing implant blooming artefact at different implant levels for all CBCT devices and image acquisition protocols.

Radar charts were used as these are depicting very well the multivariate comparisons in 2D planes, in order to demonstrate implant blooming for each CBCT device and its visualization after B&C adjustment for each device (Figs. 4 and 5). For these purposes, different axes equally distributed and uniformly drawn at 10% intervals from a common central point (0%) were created. The lines and dots within the radar chat represent the blooming artefact (in percentage) for each CBCT device according to each imaging protocol.

## Discussion

To the best of our knowledge, this was the first study to demonstrate variability of the blooming artefact generated by a titanium implant among 13 currently available CBCT devices, considering different scanning protocols. The understanding of the visualization of such device-specific artefact can contribute to the selection of the best scanning protocols to exhibit the implant dimensions as accurately as possible. Another clinical contribution of this study was to show how B&C adjustments may reduce implant blooming visualization.

To achieve the aims of this study, the most adequate design necessarily needs standardized conditions without exposing patients to ionizing radiation. Therefore, an anthropomorphic phantom was developed specifically for this project in order to avoid variations related to different individuals and, also, to enable the acquisition of multiple scans. A dental implant was placed in the mandible of that phantom and scanned with CBCT to allow for CBCT-based measurements of implant diameter, which was compared to a reference image achieved using an industrial CT scanner. Interestingly, our results showed that only a few devices presented similar perceived implant diameters when compared with the reference image. Furthermore, the severity of the blooming artefact was significantly lower after the adjustment of B&C for most of the CBCT devices.

The assessment of the blooming artefact on the present study was based on diameter measurements at five levels of a dental implant. Alternatively, we could have automatically segmented the implants to determine the resulting cross-sectional area or the entire implant volume; however, this approach would have been compromised by the inherent grey value variability between CBCT devices (i.e., unreliability of Hounsfield unit calibration) [17] as well as the varying contrast resolution. This limits the applicability of thresholding for segmentation; it has been shown that a custom segmentation method for implants yields more accurate results than a universal approach [10]. It is important to consider that during the pilot study, the authors realized that it would have been difficult to establish a reproducible threshold level in this study because some CBCT devices tend to exclude the implant threads and some tend to include the threads and the spaces between them in the segmented image, which would have resulted in under- and overestimation of the volume, respectively. Another approach used in a previous study is to quantify the severity of artefacts by means of the standard deviation of grey values within a region of interest, which can be normalized to the effective bit depth of the scan and does not rely on the mean grey value [5]. However, such an approach requires the use of a homogeneous phantom, which is different from the present study and from a clinical condition. Although the aforementioned study found a greater visualization of the blooming artefact for larger FOV sizes, the present study observed it only for a few devices.

Most of the CBCT images acquired on devices adjusted to a small-FOV high-resolution protocol did not show a significant increase in implant diameter compared to the reference image. Conversely, the implant diameter was overestimated for almost all images acquired with the small-FOV standard protocol. This increase may be related to some factors, such as spatial resolution (i.e., partial volume effect and other sources of blurring), contrast resolution (e.g., histogram distribution), filter and cut-off value used during reconstruction, beam hardening, and photon starvation [3, 4, 12]. Also, when comparing the medium FOV with small-FOV protocols at standard resolution, in a few cases, the medium FOV performed better and this can be possibly attributed to the reduced interference from the exomass, which are the structures located outside of the FOV but still between the X-ray source and the receptor [18].

Our study also demonstrated a decrease of blooming artefact after adjusting B&C for almost all CBCT devices. This decrease is related mainly to the high-contrast resolution, which permits a better distribution of the grey values, and secondarily to the partial volume effect. The lack of similar studies with such purpose precludes a direct comparison of results. However, a previous study demonstrated less blooming and, consequently, higher capability of detecting implant-abutment misfit on CBCT after B&C adjustment [11]. Some previous studies have demonstrated that the artefacts due to the presence of the implant impair the evaluation in case of dehiscence and fenestration, and that the overestimation of implant diameter may lead to an underestimation of buccal bone thickness [19–23]. It should be emphasized that our study did not intend to evaluate the influence of the blooming artefact on cortical bone visualization or diagnostic accuracy. Further studies are necessary to verify the impact of such adjustments for performing diagnostic tasks.

For the present study, clinically applicable protocols were used. Selection of these specific protocols was done considering daily practice situations. Moreover, the tested CBCT devices provide other tools, such as artefact reduction algorithms (e.g., NewTom VGi evo) and dual-energy scanning (e.g., Spectral Dental CBCT), in order to reduce artefacts; however, further studies should be undertaken to test the outcome of such applications.

Several measures have been taken to prevent biases related to the measurement method, including a well-established and accurate image registration method that was subsequently checked visually. This method assured that the measurements were performed at the same level after image registration. Additionally, measurements performed on CBCT images acquired with similar voxel sizes were compared to the reference image. Therefore, we do not believe that image registration significantly impacted metrical differences.

One inherent limitation to this ex vivo study is the absence of patient-related motion artefacts frequently observed on CBCT scans. Additionally, this study objectively demonstrated differences related to implant blooming artefact amongst CBCT devices, scanning protocols, and image adjustments, yet the present results should be further investigated by a clinical study on peri-implant diagnosis for various bone types, implant materials, designs, and dimension.

## Conclusion

Visualization of implant blooming artefact differed between CBCT devices and scanning protocols with an increase in the implant diameter ranging from 0.27 to 1.04 mm depending on the CBCT device. Most of the CBCT devices benefited from B&C adjustments to reduce blooming.

## Data Availability

The datasets used and analysed during the current study are available from the corresponding author on reasonable request.

## Abbreviations

CBCT: Cone-beam computed tomography
B&C: Brightness and Contrast
FOV: Field of view
CT: Computed tomography
PMC: Partial machined collar
NP: Narrow platform
ICC: Intraclass correlation coefficient
GLM: General linear mixed models
